# Focused evaluation of the roles of macrophages in chimeric antigen receptor (CAR) T cell therapy associated cytokine release syndrome

**DOI:** 10.20892/j.issn.2095-3941.2021.0087

**Published:** 2021-09-28

**Authors:** Hanfei Guo, Lei Qian, Jiuwei Cui

**Affiliations:** 1 Cancer Center, the First Hospital of Jilin University, Changchun 130021, China

**Keywords:** Chimeric antigen receptor, CAR T cells, cytokine release syndrome, macrophage

## Abstract

Cytokine release syndrome (CRS) is a major obstacle to the widespread clinical application of chimeric antigen receptor (CAR) T cell therapies. CRS can also be induced by infections (such as SARS-CoV-2), drugs (such as therapeutic antibodies), and some autoimmune diseases. Myeloid-derived macrophages play key roles in the pathogenesis of CRS, and participate in the production and release of the core CRS cytokines, including interleukin (IL)-1, IL-6, and interferon-γ. In this review, we summarize the roles of macrophages in CRS and discuss new developments in macrophage activation and the related mechanisms of cytokine regulation in CRS.

## Introduction

Chimeric antigen receptor (CAR) T cell therapy relies on genetic engineering to produce novel T cells that can more efficiently recognize and eliminate targeted tumor cells. CAR T cells targeting CD19 have become the standard treatment for recurrent or refractory acute B lymphoblastic leukemia and diffuse large B cell lymphoma^1–3^. Unfortunately, CAR T cell therapy can lead to severe cytokine release syndrome (CRS). CRS is more likely to occur in patients with greater tumor burden^4,5^ and/or quantities of received CAR T cells^2,6^. The overall incidence of CRS is 70%–100% across clinical studies^7,8^, and the incidence of severe CRS is approximately 10%–50%^9,10^. Therefore, studying the mechanisms of CRS is critical to preventing CAR T related illness and improving therapeutic efficacy.

Under normal circumstances, cytokines exhibit a short half-life to avoid affecting areas outside the site of inflammation. However, sustained production and elevated peripheral circulating cytokine levels may lead to systemic effects resulting in collateral damage to crucial organs; this damage may outweigh the direct benefits of the immune response. CRS, also known as a cytokine storm, is a life-threatening systemic inflammatory syndrome involving elevated levels of various cytokines and immune cells, systemic inflammation disorders, and multiple tissue dysfunction, which may lead to multiple organ failure^11^. The term “cytokine storm” was first proposed by Ferrara in 1993 to describe the immune response underlying graft *vs.* host reaction in patients undergoing hematopoietic stem cell transplantation^12^. Later, many other diseases with similar clinical pathophysiological characteristics were described to involve cytokine storms, including complications associated with some autoimmune diseases [such as macrophage activation syndrome (MAS), seen in systemic juvenile idiopathic arthritis], immunotherapy (therapeutic antibodies or transplantation)^13^, infections with microbes (toxic shock syndrome, SARS coronavirus, and COVID-19)^14^, and exposure to organic pollutants^15^. CAR T cell therapy associated CRS is generally thought to be triggered by the killing effects of CAR T cells, which induce the activation of macrophages, dendritic cells, other immune cells, and endothelial cells within the tumor environment. Once activated, these cells release pro-inflammatory cytokines, thus leading to persistent fever, blood system disorders, and ARDS^16,17^.

CRS is a complex cascade of multiple cytokines and chemokines released by the immune system in response to pathogenic substances. Studies on the mechanism of CRS have suggested that macrophages are the key cell mediators regulating pathogenesis during CRS^18^. Findings in a mouse model have confirmed that during CAR T cell therapy associated CRS, dendritic cells (DCs) and macrophages accumulate in the tumor microenvironment, and the number of macrophages in the bone marrow increases, whereas the numbers of other blood cells do not change significantly; therefore, the number of macrophages significantly increases during CRS^19^. Surface labeling analysis of cytokine-secreting cells has demonstrated that ly6C(high) macrophage cells, a pro-inflammatory lineage of monocyte-macrophages^20^, are the primary source of cytokines in CRS^19^. In addition, macrophages and endothelial cells produce large amounts of cytokines, such as interleukin (IL)-6, which activate T cells and other immune cells and form a positive feedback loop that induces the release of more cytokines and chemokines^21^. Macrophages are also known to be at the core of infection-related CRS. After recognizing viral invasion, macrophages initiate chemotaxis and recruit other immune cells by secreting the acute phase-responsive cytokines IL-6, tumor necrosis factor (TNF)-α, IL-1β, and interferon type 1 (in the innate immune system)^22^. Therefore, elucidating the mechanism underlying macrophage mediated regulation of CRS, and finding effective intervention methods based on this mechanism, has become a critical component of cell based therapeutic research. In this review, we summarize progress in mechanistic research on the pathogenesis of CRS, with the aim of providing a reference for the prevention and treatment of CAR T cell therapy associated CRS.

## Macrophage-associated CRS cytokines

The expression of many important CRS cytokines has been associated with the dysfunction of macrophages; some are excessively secreted by macrophages, including IL-1, IL-18, IL-6, and TNF, whereas others lead to the recruitment and activation of macrophages, including granulocyte-macrophage colony-stimulating factor (GM-CSF), monocyte chemo-attractant protein-1 (MCP-1), and interferon (IFN)^14,19,22,23^.

### IL-1

IL-1, the primary regulator of local and systemic inflammation reactions, is commonly encoded by 2 genes (IL1A and IL1B), both of which bind the IL-1 receptor and activate nuclear factor (NF)-κB and other inflammatory cell signaling pathways^24^. Low local concentrations of IL-1 play important roles in the immunomodulation and stimulation of antigen-presenting cells and T cells, which promote the proliferation and secretion of B cells. Overactivation of IL-1 can lead to an “inflammatory waterfall” effect causing downstream secretion of inflammatory cytokines and systemic clinical reactions such as fever and cachexia^25,26^. IL-18, a member of the IL-1 superfamily, has been associated with human hemophagocytic lymphohistiocytosis (HLH) and systemic onset juvenile inflammatory arthritis^27^. IL-18, in cooperation with IL-12 or IL-15, stimulates T cells and NK cells, and induces IFN-γ secretion, thus promoting Th1 type inflammation^28^.

IL-1 and IL-18 are produced mainly by DCs and activated monocyte-macrophages^19,25,29,30^. Pro-IL-1 β and pro-IL-18 are stored in macrophages and are activated through cleavage by caspase-1^26^. CAR T cell therapy often induces the macrophage classical pyroptosis pathway^17^, which results in the activation of the inflammasomes. The inflammasomes in turn cleave pro-caspase-1 and consequently induce caspase-1 mediated cleavage of pro-IL-1β and pro-IL-18 into mature IL-1β and IL-18, respectively, and facilitate their release to the extracellular environment^31^. In another non-canonical pyrolysis activation pathway, LPS directly binds capase-4/5/11, whose activation leads to Pannesin-1 activation and the external release of K^+^, thereby activating NLRP3 inflammasomes and ultimately resulting in IL-1β maturation and release^32,33^.

Although T cells produce small amounts of IL-1, the primary source of IL-1 in CAR T cell therapy associated CRS remains macrophages, as demonstrated by several evaluations in a related mouse model^19^. During CRS, IL-1 is produced many hours before IL-6, and because IL-1 induces the secretion of IL-6 and soluble IL-6 receptor (sIL-6R), the release of IL-1 from monocytes and macrophages in the peripheral circulation has been hypothesized to be the initiating event in CRS^34^. *In vivo* studies have confirmed that macrophage activity determines the severity of CRS, and that the IL-1 receptor antagonist anakinra decreases CAR T cell therapy associated CRS mortality^29,34^.

IL-18 may acts upstream of IL-1 and IL-6, and elevated blood IL-18 concentrations have been associated with disease activity in sHLH and MAS^27^. Evaluating the effects of secondary MAS after severe CRS induced by CAR T cell therapy is difficult because some of the diagnostic criteria overlap. A recent study by the European Society for Blood and Marrow Transplantation (EBMT) has reported a 3%–4% incidence of sHLH/MAS after CAR T cell therapy^35^. However, IL-18 has been demonstrated to enhance the anti-tumor capacity of CAR T cells *via* an IL-18R independent route but not to increase the risk of CRS associated with CAR T cell therapy^28^.

### IL-6

IL-6 has notable pro-inflammatory properties. During CRS, IL-6 and its downstream effector molecules play key roles in the occurrence of clinical symptoms and are the most important cytokines in CRS pathogenesis. High levels of IL-6 can lead to endothelial activation, hypotension, activation of the complement system, a coagulation cascade, and subsequent dissemination of intravascular coagulation, pulmonary dysfunction, myocardial dysfunction, and other pathophysiological processes^22,36^. IL-6 signal transduction can be mediated both by cis and trans signaling involving the gp130 receptor, as well as the JAK/STAT3, RAS/RAF, P13K/AKT/mTOR and MAPK–ERK pathways^37^. Gp130 is a common receptor and signal transducer of the IL-6 cytokine family. In cis signal transduction, IL-6/gp130 complexes bind the membrane-bound IL-6 receptor, whose expression is limited to immune cells. In trans signaling, the IL-6/sIL-6R complex is activated and forms a gp130 dimer on the cell surface. This dimerization in turn activates a variety of gene transcription activities mediated by JAK/STAT3 signaling, thus leading to increased secretion of a series of inflammatory factors, including MCP-1, IL-8, and additional IL-6^38^. It also increases the expression of vascular endothelial growth factor and decreases the expression of E-cadherin, thereby increasing vascular permeability and the loss of vascular stress in patients^22^. IL-6 also induces the migration of neutrophils and fibroblasts to lung epithelial cells, and consequently leads to increased collagen and fibrin deposition, and possibly lung tissue damage^22^. Cytokines initially produced by CRS, such as IL-1, IL-18, TNF-α, and IL-6 itself, strongly induce IL-6 secretion in macrophages; consequently, a positive feedback loop forms and reinforces the inflammatory response^14,39–42^.

In CAR T cell therapy associated CRS, IL-6 is produced by active T cells^43^ and endothelial cells, as well as monocyte-macrophages, the major source of this protein^44^. Norelli et al.^34^ have confirmed that monocytes are the main source of IL-6 in CRS in a newborn humanized NSG mouse model, and the depletion of monocytes or the use of the IL-6 receptor antagonist tocilizumab prevents CRS-related symptoms *in vivo*. Giavridis et al.^19^ have also demonstrated that the severity of CRS after CAR T cell infusion is closely associated with the level of IL-6 produced by macrophages in SCID mice. In a study of patients receiving CAR T cell treatment, elevated IL-6 levels have been found to be associated with grade 4 CRS^7^. IL-6 levels rise rapidly over the first 3–10 days after CAR T cell injection, reaching a peak 1 to 2 days before the number of CAR T cells peaks in the treated model^45^. In addition, elevations in IL-6 expression are much longer lived than those of the other cytokines, such as IL-1^36^, thus further supporting the hypothesis that IL-6 is critical in CAR T cell therapy associated CRS.

### TNF-α and IFN

TNF-α is primarily produced by monocytes and macrophages, and has a pro-inflammatory effect similar to that of IL-6 in CRS. TNF-α induces macrophage mediated secretion of plasminogen, which in turn leads to fibrinogen degradation, dissemination of intravascular coagulation, and eventually multiple organ dysfunction^46^. TNF-α also strongly activates the NF-κB signaling pathway and downstream inflammation-related genes, thus resulting in influenza-like symptoms accompanied by fever, fatigue, diarrhea, vascular leakage, and myocardial and lung injury^47^, and eventually leading to CRS^48^. Elevated TNF-α correlates with CRS severity^7,14^.

The mammalian IFN proteins can be divided into 3 types. Type I IFN (IFN-α/β), an important effector molecule involved in antiviral immunity, is produced by the innate immune cells (mainly macrophages). After contact with specific antigens, the macrophage surface or internal receptor (such as Toll-like receptor, NOD-like receptor, RIG-I receptor, or cGAS) transmits signals through various intracellular signaling molecules (such as MAVS, STING, TBK, or IKK) and activates the transcription factor IRF3/7, which subsequently initiates the expression of IFN-α/β^49^. Upregulation of type I IFN pathways is associated with CRS^50^. Overexpression of type I IFN enhances TNF/IL-1β-driven inflammation^51^ and the further activation of monocytes and macrophages^52^, thereby worsening the disease. Type II IFN (IFN-γ) activates macrophages, thus inducing inducible nitric oxide (iNOS) production, promoting nitric oxide synthesis, enhancing antigen processing, and inducing epithelial cell death^53^. IFN-γ can cause fever, chills, headache, dizziness, and fatigue. An *in vivo* study using a mouse model has confirmed the synergistic effects of TNF-α and IFN-γ in inducing cell death, including apoptosis, pyroptosis, and necrosis. Inhibition of TNF-α and IFN-γ decreases the risk of CRS associated mortality *in vivo*^47^; therefore, this pathway might be leveraged to develop a novel therapeutic strategy for CRS.

### GM-CSF

GM-CSF, also known as colony stimulating factor-2 (CSF-2), promotes the proliferation and differentiation of myeloid hematopoietic progenitors, particularly macrophages and eosinophils^54^. GM-CSF is mainly secreted by T cells, and high GM-CSF expression has been shown to be common in CRS after CAR T cell therapy in a mouse model^19,55^. The finding GM-CSF knock-out in CAR T cells decreases the level of GM-CSF *in vivo*^55,56^ suggests that CAR T cells may be the main source of GM-CSF in the pathogenesis of CAR T cell therapy associated CRS. GM-CSF induces monocyte-macrophage differentiation to M1 macrophages, with a phenotype producing inflammatory cytokines^57^. Inhibition of GM-CSF with a monoclonal antibody has been shown to decrease the expression of several cytokines, including IL-2, IL-1Ra, IL-6, MCP-1, and IL-8a, in a dose-dependent manner^56^. This inhibition also decreases CRS-induced weight loss *in vivo*^55^. GM-CSF receptors are highly expressed in brain glial cells, brain macrophages, and astrocytes, and these nerve cells also respond to inflammation after CAR T cell therapy, thus resulting in nerve-related adverse reactions^58^. Furthermore, blocking GM-CSF has no effect on the expression of the main T cell cytokines and does not affect the efficacy of CAR T cell therapy^56^. Therefore, GM-CSF may be a potential target for improving the design of CAR T cells, and blocking GM-CSF is also a potential strategy for treating CAR T cell therapy associated CRS.

### MCP-1

MCP-1, also called CCL2, is a key molecule in the recruitment and migration of macrophages, and its upregulation may lead to MAS^59^. Constitutive activation of NF-κB in tumor cells leads to elevated expression of MCP-1 in these tissues^60^. The production of MCP-1 in many tumor cell lines is upregulated by stimulation with IL-1, IL-6, TNF-α, or transforming growth factor-β^61,62^. Elevated MCP-1 is also associated with CRS in CAR T cell therapy. A study of 133 patients treated with CD19 CAR T cells has shown that patients with grade ≥ 4 CRS also have higher concentrations of MCP-1 and macrophage inflammatory protein (MIP)-1β^7^.

## Macrophage activation in CAR T cell therapy associated CRS

**Figure 1:**
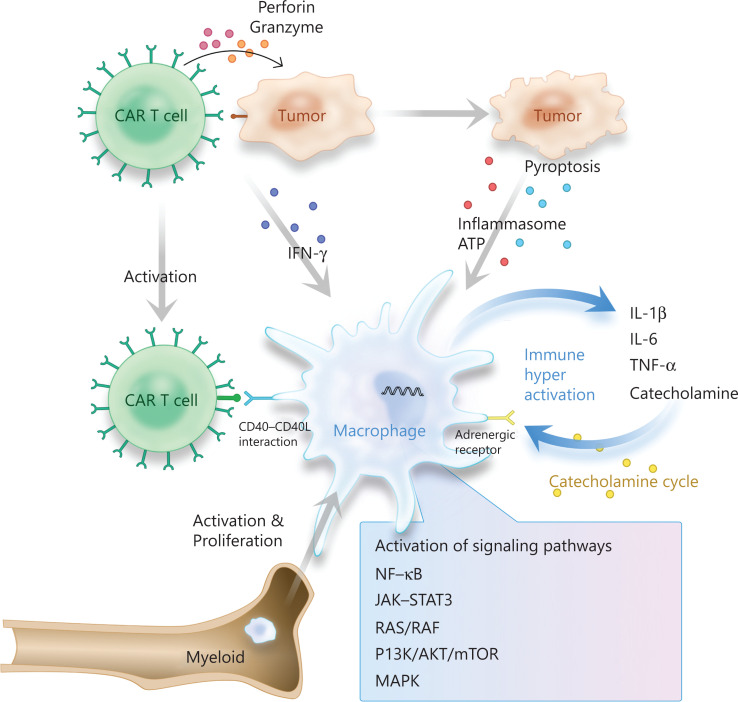
Macrophage activation in CAR T cell therapy associated cytokine release syndrome.

Macrophages in the tumor microenvironment play a key role in CRS pathogenesis. In contrast to infection-related CRS, in which viruses or pathogens directly infect macrophages, in CAR T cell therapy, CAR T cells activate macrophages through several routes including direct interaction, tumor cell pyrogenesis, and the release of ATP and inflammasomes. In addition, positive feedback from catecholamine secretion after adrenoceptor activation has also been found to stimulate macrophages and potentially lead to the continuous activation of macrophages in the tumor microenvironment (**Figure 1**).

GSDMD proteins belong to the Gasdermin family, whose N-terminal domains are involved in membrane perforation and induced pyrogenesis. GSDMD, the common substrate of caspase-1 and caspase-4/5/11, is normally self-inhibited. After being cleaved by the caspase proteins, it releases its N-terminal domain with membrane perforation activity, thus leading to cell swelling and rupture^63^. CAR T cells activate GSDME cleavage in tumor cells by releasing perforin/granzyme B, thereby inducing tumor cell pyrogenesis. Inflammasomes released by tumor cells activate the caspase1-GSDMD pathway in macrophages and consequently lead to the upregulation of various inflammatory cytokines including IL-1 and IL-6 in macrophages^17^.

Direct interaction mediated by CD40 L-CD40 may also occur between CAR T cells and monocyte-macrophages. CD40 L, also known as gp39 and TNF associated activation protein (TRAP), are primarily expressed in activated T cells. CD40 is expressed in B cells, activated monocytes/macrophages, DCs, hematopoietic progenitor cells, and endothelial cells, among others. The CD40 L-CD40 signal regulates the activity of various non-receptor tyrosine protein kinases and transcription factors including NF-κB^64^, and the activation of CD40 L-CD40 signaling in macrophages directly induces the expression and secretion of IL-6^65^. When CAR T cells expressing mCD40 L are injected into an NSG mouse model, CD40 L-CD40 interaction between the CAR T cells and monocyte-macrophages is associated with CRS, and this interaction correlates with the severity of the CRS^19^, thus suggesting that CAR T cells may regulate the production of cytokines in macrophages through CD40L-CD40 signaling. However, human CD40L (hCD40L) cannot directly bind mouse CD40 (mCD40)^66^, and the absence of CD40L-CD40 interactions does not appear to affect CRS onset in the mouse model^19^. Whether this direct interaction is a mechanism underlying CRS during CAR T cell therapy remains unknown and therefore is likely to be closely evaluated in the future.

When macrophages are activated, they in turn activate the adrenergic receptors. The α1-adrenoceptor dependent signal results in activation and release of a variety of downstream cytokines, including IL-6, TNF, MIP-2, and catecholamines, which enhance inflammatory injury^67^. These cytokines further interact with macrophages, thereby inducing the production and release of more catecholamines, and forming a positive feedback loop^21^. Interactions between hCART19-cells and Raji cells have been found to result in the release of catecholamines and cytokines (such as IL-2, TNF, IFN-γ, and MIP-1β) from macrophages, a response correlating with CRS severity^68^.

## Biomarkers of CRS

CRS is caused by the dysregulated proliferation of inflammatory cytokines. Circulating cytokines can be used as biomarkers for CRS diagnosis and prognosis. After infusion with CAR T cells, the levels of serum cytokines change with changes in CAR T cell expansion and tumor load, and generally increase rapidly for the first 5–10 days. Subsequently, the expansion of CAR T cells slows, the tumor load decreases, and the levels of cytokines decrease^3^.

Many cytokines increase during CRS, including IL-1, IL-18, IL-6, IL-10, IL-15, IFN-γ, MCP-1, Ang2/Ang1, GM-CSF, TNFRp55, and MIP-1β^69–72^. However, because only the baseline level of IL-6 before treatment is correlated with CRS, this protein may have good predictive value for CRS. In a clinical study involving 74 patients treated with CAR T cell therapy, the incidence of grade ≥ 3 CRS after CAR T cell infusion was 56% in patients with an IL-6 level of ≥ 40 pg/mL, and the mortality rate of these patients within 90 days was as high as 89%^70^.

An increase in serum acute reactive protein C (CRP) and ferritin production has also been observed in patients with CAR T cell therapy associated CRS^73^, and the peak ferritin value has been found to correlate with the severity of CRS^13,74^. IFN or pathogens activate macrophages to release high levels of ferritin; consequently, this increase in ferritin may be directly associated with an increase in macrophage activation^75^. Elevated ferritin has also been observed in children with MAS^76^. However, CRP and ferritin are poorly specific in predicting CAR T cell therapy associated CRS, and the positive predictive value of elevated ferritin after CAR T treatment in predicting the severity of CRS is less than 50%^71^. Studies have shown that MCP-1 combined with a fever ≥ 38.9 °C has high sensitivity and specificity for predicting severe CRS within 36 h of CAR T cell injection^7^. In short, the exploration of biomarkers to predict CRS remains in progress, and effective markers for predicting CRS in response to CAR T cell therapies remain lacking.

## Prospects of macrophage-centered CRS prevention and treatment

### Prevention of CAR T cell therapy associated CRS

Adjusting the dose of CAR T cells on the basis of tumor load^2,4–6^ or changing the structure of the CAR T cells^77,78^ can effectively prevent severe CRS. The costimulatory ability and antigen-binding characteristics of the CAR structure may also affect the risk of CRS^79^. In second generation CAR structures, the addition of the CD28 costimulatory signal domain increases the incidence of CRS, whereas CAR structures with a 4-1BB domain show a slight propensity for CRS^80^. In addition, the degree of inhibition of bone marrow hematopoiesis caused by preconditioning is also associated with the intensity of the CRS associated with CAR T cell therapy^81^.

Cytokine analysis may effectively predict the occurrence of CRS. Patients at high risk of CRS are given chemotherapy in advance to decrease the tumor load; receive an appropriately reduced number of CAR T cell infusions; and are treated with an array of other preventive measures before being closely monitored for symptoms of CRS during treatment. These interventions are designed to retain therapeutic efficacy while preventing and alleviating CRS. Vital signs should be monitored every 2 h after CAR T cell transfusions, and routine blood examinations and blood biochemical indexes, such as IL-6, ferritin, and CRP, should be verified daily.

### Treatment of CAR T cell therapy associated CRS

Grade 1 CRS requires close monitoring, evaluation for infection, and symptomatic support, and most patients recover. Monoclonal antibodies against the IL-6 receptor (tocilizumab) and corticosteroids are usually recommended for patients with grade 2–4 CRS. Corticosteroids effectively inhibit CRS and are the first choice in controlling CRS toxicity. Their addition does not affect the efficacy of CAR T cell therapy^82^. Tocilizumab quickly reverses the symptoms of CRS^83^, and studies have shown that treatment with tocilizumab facilitates CRS control in 53%–69% of cases^84^ without any adverse effects on the immune system^5^. However, tocilizumab cannot cross the blood-brain barrier, and the addition of steroids has been suggested to help control neurotoxicity^85^. A variety of treatments targeting the key cytokines in CRS are also being explored; however, to date, only the IL-6 antibody tocilizumab has been approved by the FDA for the treatment of CRS^84^. Siltuximab, a monoclonal antibody with a higher affinity for IL-6 than tocilizumab, targets IL-6R and can effectively remove active IL-6 from circulation, thus preventing IL-6 mediated immune activation and decreasing CRS severity^86^. Sarilumab is a monoclonal antibody that blocks IL-6R, and has been approved by the U.S. Food and Drug Administration (FDA) for the treatment of rheumatoid arthritis^87^. However, an open-label cohort study has not found that sarilumab improves systemic hyperinflammation in patients with severe COVID-19^88^. The IL-1 receptor antagonist anakinra is an effective treatment for CRS associated with secondary HLH^76^, and its safety profile for CAR T cell therapy associated CRS remains under exploration^29^. Inﬂiximab targets TNF-α, and has been used for the management of immunotherapy-related enterocolitis^89^. GM-CSF is an important regulatory factor of monocyte-macrophages in the inflammatory response, and the anti-GM-CSF monoclonal antibody, lenzilumab decreases the effects of cytokines and neurotoxicity in a dose-dependent manner^21,56^. Well-designed clinical trials are needed to evaluate treatments that block cytokines in managing CRS.

Regulating immune cells or bystander cells is a new strategy for controlling CRS. Studies have shown that macrophages secrete catecholamines and are also induced by catecholamines, which in turn stimulate the production of cytokines. The compound α-methyltyrosine, an inhibitor of tyrosine hydroxylase, the key rate-limiting enzyme in catecholamine synthesis, may attenuate CRS by limiting the activity of the catecholamine pathway in macrophages^67^. *In vitro* studies have shown that atrial natriuretic peptide inhibits CRS, by limiting the synthesis of catecholamines without affecting the killing effect of CAR T cells^68^. iNOS is mainly expressed in activated macrophages^90^ and is induced by IL-6 and IL-1^91,92^. Increased production of nitric oxide can lead to common clinical manifestations of CRS, such as vasodilation and hypotension. *In vivo* studies have shown that macrophages are the primary source of iNOS after CAR T cell therapy in mice. Treatment with the iNOS inhibitor L-N6-(1-Iminoethyl)-lysine (L-NIL) relieves the CRS and improves the survival rate in these animals^19^.

## Summary and future perspectives

The pathogenesis of CRS involves complex and interrelated networks of various immune cells, signaling pathways, and cytokines, all of which require further evaluation. The establishment of a mouse model is the primary challenge in exploring the mechanism of CRS. Immunodeficient mice are the first choice in constructing tumor models for the study of CAR T cell therapy associated CRS. However, because CRS is an overreaction of the immune system, immunodeficient mice may not be able to fully recapitulate CRS after CAR T cell therapy. The species differences between humans and mice (in immune system composition) and the intraspecies differences in genetic background among mouse strains all influence the establishment of CRS models. For example, CRS in the SCID mouse model is more severe than that in the NSG mouse model^19^. Further studies on the mechanism of CRS are necessary to decrease the incidence of CRS, broaden the therapeutic applications of CAR T cell therapy, and provide a theoretical basis for individualized treatment by using predictive biomarkers for CRS.

CAR T cell therapy provides hope for a variety of intractable malignant tumors; however, CAR T cell therapy associated CRS severely affects the clinical application of this treatment. This article reviewed the research progress in macrophages and their effects on the pathogenesis of CRS, an important adverse reaction to CAR T cell therapy. CRS-related macrophages are derived mainly from myeloid cells^19^ and are the primary source of the core CRS cytokines (such as IL-1, IL-6, and TNF)^14,19,22,23^. In view of the important roles of macrophages in CRS, we have summarized several potential macrophage-based treatments designed to interfere with CRS. In short, understanding the effects of macrophages on CRS will enable better understanding of CAR T cell therapy associated CRS and may broaden the application of CRS therapy to benefit more patients in the future.
